# Supplementary material for: Pharmacological inhibition of HDAC6 improves muscle phenotypes in dystrophin-deficient mice by downregulating TGF-β via Smad3 acetylation
Source: Nat Commun. 2022 Nov 19;13:7108. doi: 10.1038/s41467-022-34831-3 (PMC9675748; doi:10.1038/s41467-022-34831-3)
Supplement: Supplementary file 1 — Supplementary Information [file 41467_2022_34831_MOESM1_ESM.pdf]

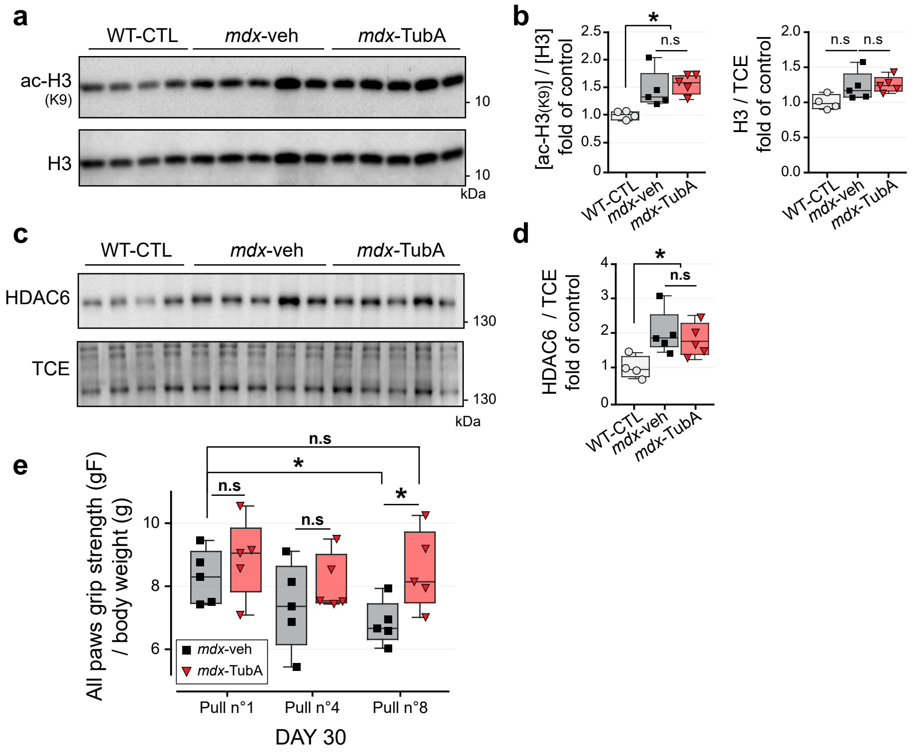

Supplementary Figure 1 | HDAC6 inhibition *via* TubA treatment does not regulate histone H3 acetylation and HDAC6 expression and maintains grip strength in *mdx* mice. a, b, To evaluate levels of histone H3 acetylation (ac-H3K9) and HDAC6 in TA muscles, Western blot analysis (**a, c**) and quantifications (**b, d**) were performed. First, the membrane was probed with the ac-H3(K9) antibody and then, striped and re-probed with the H3 antibody (**a**). Quantifications of acetylated histone H3 protein level (**b**, n=4 or 5 mice per group) was normalized to histone H3 whereas HDAC6 protein level (**d**, n=4 or 5 mice per group) was normalized to the total proteins in the cell extract (TCE). **e**, Grip strength was measured on a grid over 8 pulls normalized to body weight (n=5 mice per group). (**b, d, e**) Whiskers min to max; the line in the middle of the box is plotted at the median. *, $P < 0.05$; n.s, not significant, $P > 0.05$; Mann-Whitney *U* test; kDa, relative molecular weight in kiloDalton.

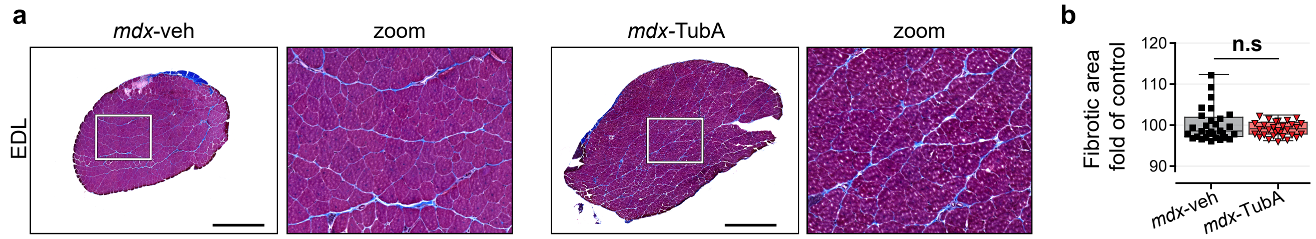

Supplementary Figure 2 | HDAC6 inhibition prevents the formation of fibrotic hotspots in mdx EDL muscle. a, Cross-sections of entire EDL muscles from C57BL/10ScSn-Dmd*mdx*/J mice treated with vehicle-DMSO (*mdx*-veh) or with TubA (*mdx*-TubA) for 30 consecutive days were stained using Masson's Trichrome. Scale bars: 500 µm. **b,** Quantification of collagen content infiltration level was performed. Fibrotic area are colored in blue (n=30-33 fields counted per mouse; 3 mice per group). Scale bars: 500 µm. (**b**) Whiskers min to max; the line in the middle of the box is plotted at the median. n.s, not significant, P > 0.05; Mann-Whitney *U* test.

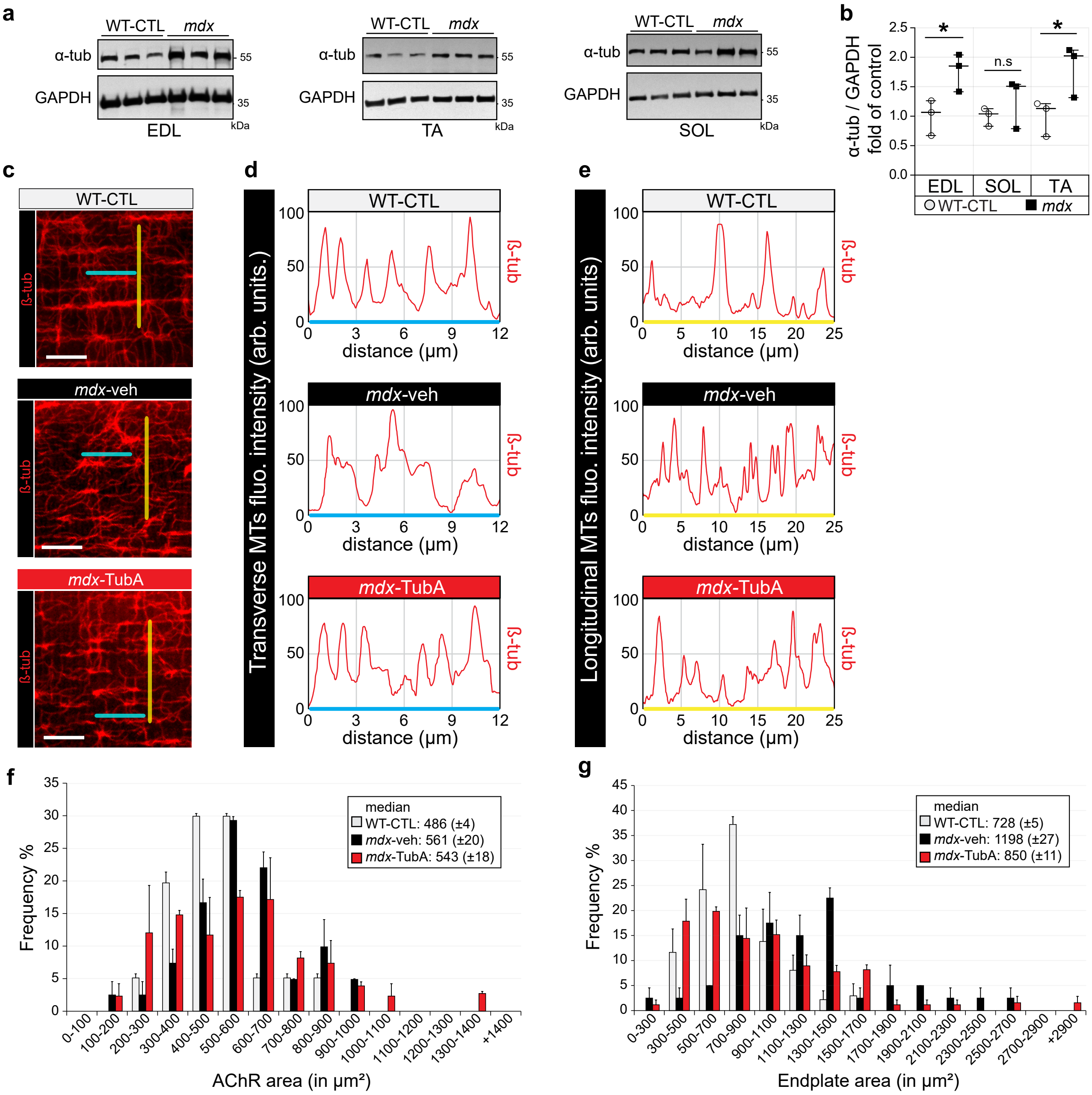

Supplementary Figure 3 | Level of α-tubulin increase in *mdx* mice and TubA regulates both MT network and NMJs in dystrophic mice. a, b, To evaluate the level of α-tubulin (α-tub) in EDL, SOL and TA of WT and *mdx* muscles, Western blot analysis (**a**) and quantification (**b**) were performed. **a**, GAPDH was used as a loading control. **b**, Data are presented as median values ± SEM (n=3 mice per group). *, P < 0.05; n.s, not significant. kDa, relative molecular weight in kiloDalton. **c**, Isolated fibers of TA from 11-wk-old C57BL/10 mice (WT-CTL) and C57BL/10ScSn-Dmd*mdx*/J mice treated with vehicle-DMSO (*mdx*-veh) or with TubA (*mdx*-TubA) for 30 consecutive days were stained with an antibody against β-tubulin (ß-tub) to label MT network (in red). Scale bars: 10 µm. **d, e**, The fluorescence intensity of β-tubulin was plotted as a function of the distance based on the blue (**d**, to visualized transverse MTs fluorescence intensity) and yellow (**e**, to visualized longitudinal MTs fluorescence intensity) line scans presented in extended data Fig. 3c. arbitrary units (arb. units). *, P < 0.05; n.s, not significant, P > 0.05; Mann-Whitney U test. Distribution of acetylcholine receptor (AChR) surface (**f**) and endplate area (**g**). The median value of each group is indicated in the boxes above the histograms. **f, g**, Data are presented as mean values ± SEM (n=45 to 70 NMJs were counted for each condition).

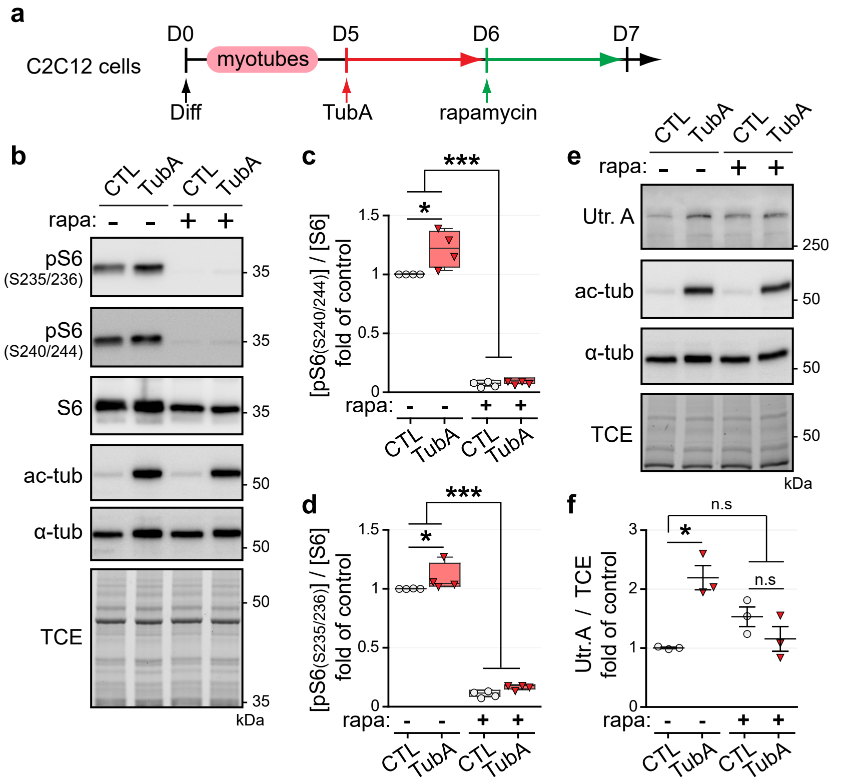

Supplementary Figure 4 | Rapamycin prevents the increase in utrophin A levels induced by TubA and HDAC6 inhibition does not prevent the inhibition of the phosphorylation of the mTOR downstream target S6. a, Schematic representation of the experimental set up. D: days. Diff: addition of the differentiation medium. **b-f**, Cells were treated either with TubA (5 µM) or DMSO (CTL, 1 µl) at day 5 and rapamycin was added or not at day 6 (Rapa-, Rapa+; 100nM). Cells were collected at day 7 for analysis by Western blot. **b**, Representative Western blots showing pS6 (S240/244), pS6(S235/236), S6, acetylated tubulin (ac-tub), and α-tubuline. Quantifications of pS6 (**c,** S240/244) and pS6 (**d,** S235/236) protein levels were normalized to S6 (n=4 independent experiments quantified). **e**, Representative Western blots showing utrophin A, acetylated tubulin (ac-tub), and α-tubuline. **f**, Quantification of utrophin A protein levels was normalized to TCE (n=3 independent experiments quantified; two-way ANOVA). Data are presented as mean values ± SEM. TCE was used as a loading control. (**e, d**) Whiskers min to max; the line in the middle of the box is plotted at the median. *, P < 0.05; ***, P < 0.001; n.s, not significant, P > 0.05; two-way ANOVA (**c, d**). kDa, relative molecular weight in kiloDalton.

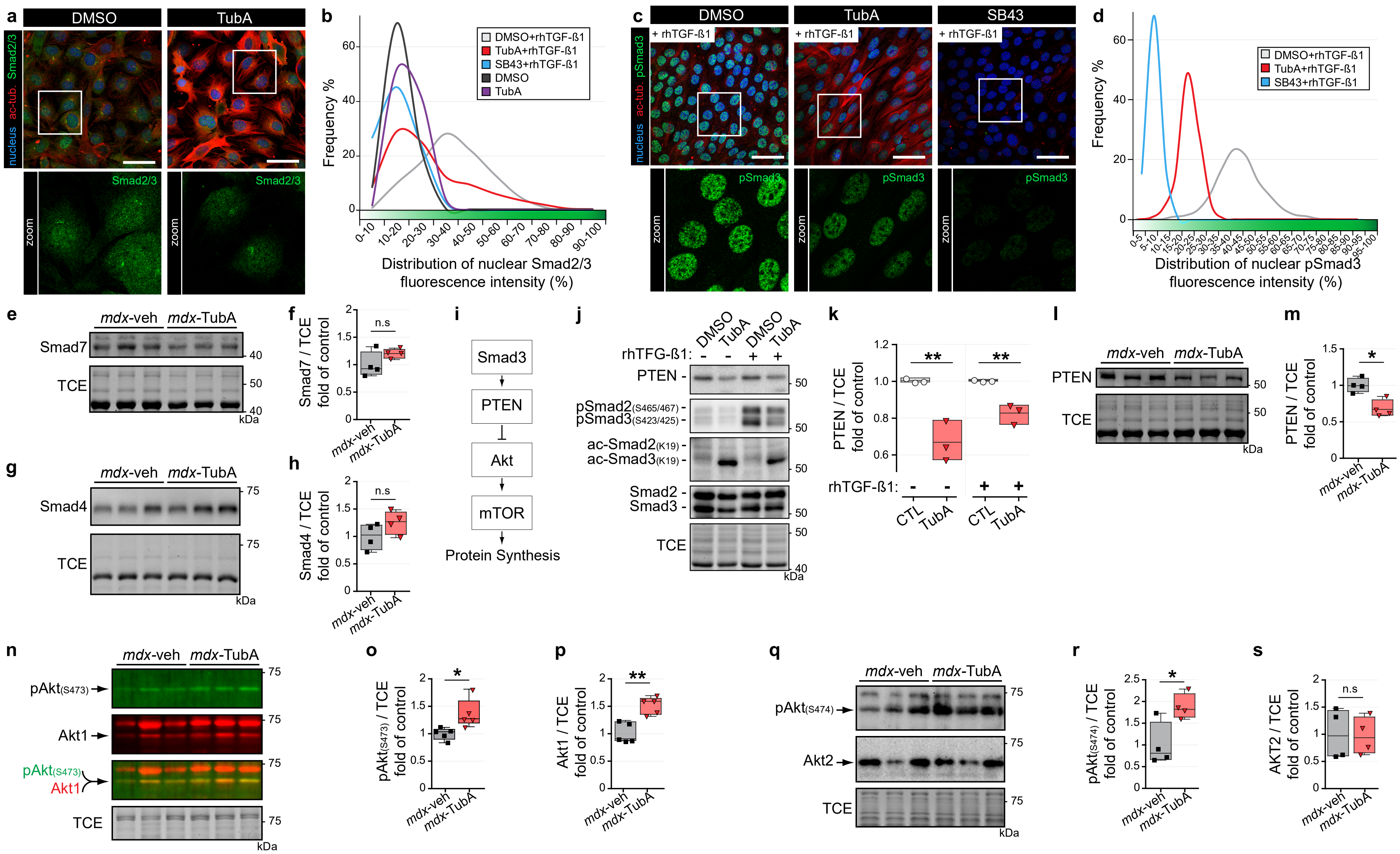

Supplementary Figure 5 | Inhibition of HDAC6 protects against TGF-β treatment and regulates PTEN/Akt signaling related to the mTOR pathway. 4-day-old C2C12 myoblasts pretreated for 24 h with either HDAC6 inhibitor (TubA, 5 μM), or selective inhibitor of TGF-β1 (SB43, 5 μM) or with DMSO (CTL, 1 μL). Then, myoblasts were treated for 30 min with rhTGF-β1 (10 ng/mL). **a**, Myoblasts were double-stained either with antibodies against Smad2/3 (**a**, in green) or against phosphorylated Smad3 (**c**, pSmad3, in green) and acetylated tubulin (ac-tub, in red). Nuclei were labeled with DAPI (in blue). Scale bars: 50 μm. **b**, Summary of graphical curves of the nuclear distribution of Smad2/3 fluorescence intensity combining all results obtained both in Fig. 5a and in extended data Fig. 5a (n=3 independent experiments quantified). **d**, Summary of graphical curves of the nuclear distribution of phosphorylated Smad3 fluorescence intensity from 2 independent experiments quantified. TA muscles from 11-wk-old C57BL/10ScSn-Dmd*mdx*/J mice treated with TubA (*mdx*-TubA) or with vehicle-DMSO (*mdx*-veh) for 30 consecutive days were analyzed by Western blot (**e**, **g**, **l**, **n**, **q**) and quantified (**f**, **h**, **m**, **o**, **p**, **r**, **s**). **i**, Schematic summary of the link between Smad3 and mTOR. **j**, Western blots were performed from 4-day-old C2C12 myoblasts. To evaluate levels of Smad7 (**f**), Smad4 (**h**), PTEN (**k**, **m**), AKT phosphorylations (**o**, **r**), Akt1 (**p**) and Akt2 (**s**) quantifications were performed (n=4-5 mice per group). Quantifications of protein levels were normalized to the total proteins in the cell extract (TCE); Mann-Whitney *U* test. (**f**, **h**, **k**, **m**, **o**, **p**, **r**, **s**) Whiskers min to max; the line in the middle of the box is plotted at the median.*, P < 0.05; **, P < 0.01; n.s, not significant, P > 0.05. kDa, relative molecular weight in kiloDalton.

Supplementary table 1: Primary Antibodies

antibody name	type	dilution		provider
		Immunostaining	Western blotting	
β-tubulin	mouse monoclonal	1:500		Sigma-Aldrich, clone TUB2.1, #T5201
acetylated tubulin	mouse monoclonal	1:200	1:2,000	Sigma-Aldrich, clone 6-11 B-1 #T7451
α-tubulin	mouse monoclonal		1:1,000	Sigma-Aldrich, clone B-5-1-2, #T6074
Histone H3 (D1H2)	rabbit monoclonal		1:1,000	Cell Signaling Technology, clone D1H2, #4499
acetyl-Histone H3 (Lys9)	rabbit polyclonal		1:1,000	Sigma-Aldrich, #07-352
Utrophin A	mouse monoclonal	1:200	1:500	Novocastra, Leica biosystems, clone DRP3/20C5, #NCL-DRP2
β-dystroglycan	mouse monoclonal	1:400	1:500	Novocastra, Leica biosystems, clone 43DAG1/8D5, #NCL-b-DG
Atrogin-1 (MAFbx)	rabbit polyclonal		1:1,000	ECM Biosciences, #AP2041
MuRF1	mouse monoclonal		1:1,000	Abcam, #ab57865
Col1A1	goat polyclonal		1:200	Santa Cruz Biotechnology, #sc-25974
CTGF	rabbit polyclonal		1:1,000	Abcam, #ab6992
mTOR	rabbit polyclonal		1:1,000	Cell Signaling Technology, #2972
Phospho-p70 S6 Kinase (Thr389)	rabbit polyclonal		1:1,000	Cell Signaling Technology, #9205
p70 S6 Kinase	rabbit polyclonal		1:1,000	Cell Signaling Technology, #9202
Phospho-S6 Ribosomal Protein (Ser240/244)	rabbit polyclonal		1:1,000	Cell Signaling Technology, #2215
Phospho-S6 Ribosomal Protein (Ser235/236) (D57.2.2E)	rabbit monoclonal		1:1,000	Cell Signaling Technology, #4858
S6 Ribosomal Protein (5G10)	rabbit monoclonal		1:1,000	Cell Signaling Technology, #2217
Phospho-4E-BP1 (Thr37/46) (236B4)	rabbit monoclonal		1:1,000	Cell Signaling Technology, #2855
Phospho-4E-BP1 (Thr70)	rabbit polyclonal		1:1,000	Cell Signaling Technology, #9455
4E-BP1	rabbit polyclonal		1:1,000	Cell Signaling Technology, #9452
Phospho-Smad2 (Ser465/467) /Smad3 (Ser423/425) (D27F4)	rabbit monoclonal		1:1,000	Cell Signaling Technology, #8828
Smad2/3 (D7G7)	rabbit monoclonal	1:400	1:1,000	Cell Signaling Technology, #8685
Acetyl-Smad2/Smad3 (Lys19)	rabbit polyclonal		1:1,000	Invitrogen, #PA5-76015
Smad3 (phospho S423/S425)	rabbit polyclonal	1:200	1:1,000	Abcam, #ab52903
laminin	rabbit polyclonal	1:200		Sigma-Aldrich, #L9393
GAPDH (HRP)	goat polyclonal		1:20,000	Abcam, #ab85760
HDAC6 (D21B10)	rabbit monoclonal		1:4,000	Cell Signaling Technology, #7612
Smad4	rabbit monoclonal		1:1,000	Cell Signaling Technology, #46535
Smad7	rabbit polyclonal		1:1,000	ABclonal, #A12343
PTEN	rabbit polyclonal		1:2,000	ABclonal, #A11528
Akt1 (phospho S473)	rabbit polyclonal		1:1,000	Cell Signaling Technology, #9271
Akt1 (2H10)	mouse monoclonal		1:1,000	Cell Signaling Technology, #2967
Akt2 (phospho S474) (D3H2)	rabbit monoclonal		1:1,000	Cell Signaling Technology, #8599
Akt2 (5B5)	rabbit monoclonal		1:1,000	Cell Signaling Technology, #2964
