# Supplementary material for: Pharmacological inhibition of HDAC6 improves muscle phenotypes in dystrophin-deficient mice by downregulating TGF-β via Smad3 acetylation
Source: Nat Commun. 2022 Nov 19;13:7108. doi: 10.1038/s41467-022-34831-3 (PMC9675748; doi:10.1038/s41467-022-34831-3)
Supplement: Supplementary file 3 — Reporting Summary [file 41467_2022_34831_MOESM3_ESM.pdf]

nature portfolio

Corresponding author(s): Laurent Schaeffer

Last updated by author(s): Oct 7, 2022

Reporting Summary

Nature Portfolio wishes to improve the reproducibility of the work that we publish. This form provides structure for consistency and transparency in reporting. For further information on Nature Portfolio policies, see our Editorial Policies and the Editorial Policy Checklist.

Statistics

For all statistical analyses, confirm that the following items are present in the figure legend, table legend, main text, or Methods section.

n/a	Confirmed	
☐	☒	The exact sample size (*n*) for each experimental group/condition, given as a discrete number and unit of measurement
☐	☒	A statement on whether measurements were taken from distinct samples or whether the same sample was measured repeatedly
☐	☒	The statistical test(s) used AND whether they are one- or two-sided<br>*Only common tests should be described solely by name; describe more complex techniques in the Methods section.*
☒	☐	A description of all covariates tested
☐	☒	A description of any assumptions or corrections, such as tests of normality and adjustment for multiple comparisons
☐	☒	A full description of the statistical parameters including central tendency (e.g. means) or other basic estimates (e.g. regression coefficient) AND variation (e.g. standard deviation) or associated estimates of uncertainty (e.g. confidence intervals)
☐	☒	For null hypothesis testing, the test statistic (e.g. *F*, *t*, *r*) with confidence intervals, effect sizes, degrees of freedom and *P* value noted<br>*Give P values as exact values whenever suitable.*
☒	☐	For Bayesian analysis, information on the choice of priors and Markov chain Monte Carlo settings
☒	☐	For hierarchical and complex designs, identification of the appropriate level for tests and full reporting of outcomes
☒	☐	Estimates of effect sizes (e.g. Cohen's *d*, Pearson's *r*), indicating how they were calculated

Our web collection on statistics for biologists contains articles on many of the points above.

Software and code

Policy information about availability of computer code

Data collection	Zeiss LSM880 microscope (Carl Zeiss) with an AiryScan1 detector equipped with a 63× 1.4-NA objective<br>Zeiss Axio Imager M2 (Carl Zeiss) upright microscope equipped with either a 63× 1.4-NA or a 10× 0.45 NA objectives<br>Zeiss Axio Scan. Z1 (Carl Zeiss) equipped with a Plan-Apochromat 20x/0.8 objective<br>AxioCam mRm CCD detector<br>ChemiDocTM MP Imaging Systems (Bio-Rad)<br>autoradiographed with X-Ray films (Fisher Scientific)<br>QPCR CFX Connect (Bio-rad)
Data analysis	GraphPad Prism 6.0 (GraphPad Software, La Jolla, USA)<br>FIJI software (ImageJ 2.0.0-rc-69/1.52n, National Institutes of Health, Bethesda, MD)<br>Image Lab software 6.0 (Bio-Rad)<br>ZEN 2 blue edition software (Zeiss, Oberkochen, Germany)<br>Zeiss AxioVision software V 4.9.1.0 (Zeiss, Oberkochen, Germany)<br>Photoshop CS5 version 7.0 (Abobe Systems, San Jose, CA, USA)<br>Adobe Illustrator version 14.0.0 (Abobe Systems, San Jose, CA, USA)

For manuscripts utilizing custom algorithms or software that are central to the research but not yet described in published literature, software must be made available to editors and reviewers. We strongly encourage code deposition in a community repository (e.g. GitHub). See the Nature Portfolio guidelines for submitting code & software for further information.

Data

Policy information about availability of data

All manuscripts must include a data availability statement. This statement should provide the following information, where applicable:

- Accession codes, unique identifiers, or web links for publicly available datasets
- A description of any restrictions on data availability
- For clinical datasets or third party data, please ensure that the statement adheres to our policy

All data generated and/or analysed during this study are included in this published article (and its supplementary information files).

Human research participants

Policy information about studies involving human research participants and Sex and Gender in Research.

Reporting on sex and gender	N/A
Population characteristics	N/A
Recruitment	N/A
Ethics oversight	N/A

Note that full information on the approval of the study protocol must also be provided in the manuscript.

Field-specific reporting

Please select the one below that is the best fit for your research. If you are not sure, read the appropriate sections before making your selection.

☒ Life sciences ☐ Behavioural & social sciences ☐ Ecological, evolutionary & environmental sciences

For a reference copy of the document with all sections, see nature.com/documents/nr-reporting-summary-flat.pdf

Life sciences study design

All studies must disclose on these points even when the disclosure is negative.

Sample size	Sample sizes were determined based on established practice and applicable standards. We did not performed statistical methods to predetermine sample size. We opted for sample sizes that are commonly used sample sizes in the field (at least 3 experiments in vitro and at least 3 mice in each group). These sample sizes are sufficient to evaluate effect of tubastatin A (Osseni, A. et al., 2020, d'Ydewalle, C. et al., 2011).
Data exclusions	No data were excluded from analysis.
Replication	Biological and independent replicate experiments were successful. In vivo expriments are replicates and were performed with indicated numbers of animals. For in vivo studies, a minimum of three biological replicates were analyzed. Each experiment in which data were quantified was performed with at least 3 replicates. Sample sizes and statistical analyses and significance levels are all indicated in the figure legends or in the method part. Each experiment in which data were quantified was performed with at least three replicates.
Randomization	All behavioral tests were performed in a blinded manner at the University of Ottawa Animal Behavior core facility. Allocation for Mdx mice groups injected either with saline+DMSO or with tubastatin A+DMSO was random.<br>For cell culture experiments, cells were split, plated, and then treated with DMSO or drugs. Because control and treatment groups were derived from the same cell line, no randomization could be performed at this point. After images acquisition and then for image analysis of cell culture, a blinded manner by randomly renaming file names with numbers was performed using the 'name_randomizer' macro in ImageJ (Osseni, A. et al., 2016).
Blinding	All behavioral tests were performed in a blinded manner at the University of Ottawa Animal Behavior core facility. The investigators were blinded to group allocation during data analysis because all images were analyzed in a blinded manner by randomly renaming file names with numbers using the 'name_randomizer' macro in ImageJ (Osseni, A. et al., 2016).

Reporting for specific materials, systems and methods

We require information from authors about some types of materials, experimental systems and methods used in many studies. Here, indicate whether each material, system or method listed is relevant to your study. If you are not sure if a list item applies to your research, read the appropriate section before selecting a response.

Materials & experimental systems

n/a	Involved in the study
☐	☒ Antibodies
☐	☒ Eukaryotic cell lines
☒	☐ Palaeontology and archaeology
☐	☒ Animals and other organisms
☒	☐ Clinical data
☒	☐ Dual use research of concern

Methods

n/a	Involved in the study
☒	☐ ChIP-seq
☒	☐ Flow cytometry
☒	☐ MRI-based neuroimaging

Antibodies

Antibodies used

β-tubulin mouse monoclonal 1:500 IF Sigma-Aldrich, clone TUB2.1, #T5201
acetylated tubulin mouse monoclonal 1:200 IF; 1:2,000 WB; Sigma-Aldrich, clone 6-11 B-1 #T7451
α-tubulin mouse monoclonal 1:1,000 WB; Sigma-Aldrich, clone B-5-1-2, #T6074
Histone H3 (clone D1H2) rabbit monoclonal 1:1,000 WB; Cell Signaling Technology, #4499
acetyl-Histone H3 (Lys9) rabbit polyclonal 1:1,000 WB; Sigma-Aldrich, #07-352
Utrophin A mouse monoclonal 1:200 IF; 1:500 WB; clone DRP3/20C5, Novocastra, Leica biosystems, #NCL-DRP2
β-dystroglycan mouse monoclonal 1:400 IF; 1:500 WB; clone 43DAG1/8D5, Novocastra, Leica biosystems, #NCL-b-DG
Atrogin-1 (MAFbx) rabbit polyclonal 1:1,000 WB; ECM Biosciences, #AP2041
MuRF1 mouse monoclonal 1:1,000 WB; Abcam, #ab57865
Col1A1 goat polyclonal 1:200 WB; Santa Cruz Biotechnology, #sc-25974
CTGF rabbit polyclonal 1:1,000 WB; Abcam, #ab6992
mTOR rabbit polyclonal 1:1,000 WB; Cell Signaling Technology, #2972
Phospho-p70 S6 Kinase (Thr389) rabbit polyclonal 1:1,000 WB; Cell Signaling Technology, #9205
p70 S6 Kinase rabbit polyclonal 1:1,000 WB; Cell Signaling Technology, #9202
Phospho-S6 Ribosomal Protein (Ser240/244) rabbit polyclonal 1:1,000 WB; Cell Signaling Technology, #2215
Phospho-S6 Ribosomal Protein (Ser235/236) (clone D57.2.2E) rabbit monoclonal 1:1,000 WB; Cell Signaling Technology, #4858
S6 Ribosomal Protein (clone 5G10) rabbit monoclonal 1:1,000 WB; Cell Signaling Technology, #2217
Phospho-4E-BP1 (Thr37/46) (clone 236B4) rabbit monoclonal 1:1,000 WB; Cell Signaling Technology, #2855
Phospho-4E-BP1 (Thr70) rabbit polyclonal 1:1,000 WB; Cell Signaling Technology, #9455
4E-BP1 rabbit polyclonal 1:1,000 WB; Cell Signaling Technology, #9452
Phospho-Smad2 (Ser465/467) /Smad3 (Ser423/425) (clone D27F4) rabbit monoclonal 1:1,000 WB; Cell Signaling Technology, #8828
Smad2/3 (clone D7G7) rabbit monoclonal 1:400 IF; 1:1,000 WB; Cell Signaling Technology, #8685
Acetyl-Smad2/Smad3 (Lys19) rabbit polyclonal 1:1,000 WB; Invitrogen, #PA5-76015
Smad3 (phospho S423/S425) rabbit polyclonal 1:200 IF; 1:1,000 WB;Abcam, #ab52903
Laminin rabbit polyclonal 1:200 IF; Sigma-Aldrich, #L9393
GAPDH (HRP) goat polyclonal 1:20,000 WB; Abcam, #ab85760
HDAC6 (clone D21B10) rabbit monoclonal 1:4,000 WB; Cell Signaling Technology, #7612
Smad4 (clone D3R4N) rabbit monoclonal 1:1,000 WB; Cell Signaling Technology, #46535
Smad7 rabbit polyclonal 1:1,000 WB; ABclonal, #A12343
PTEN rabbit polyclonal 1:2,000 WB; ABclonal, #A11528
Akt1 (phospho S473) rabbit polyclonal 1:1,000 WB; Cell Signaling Technology, #9271
Akt1 (clone 2H10) mouse monoclonal 1:1,000 WB; Cell Signaling Technology, #2967
Akt2 (phospho S474) (clone D3H2) rabbit monoclonal 1:1,000 WB; Cell Signaling Technology, #8599
Akt2 (clone 5B5) rabbit monoclonal 1:1,000 WB; Cell Signaling Technology, #2964 v

Donkey anti-Mouse IgG (H+L) Highly Cross-Adsorbed Secondary Antibody, Alexa Fluor™ 488, 1:1000 IF, Invitrogen, # A-21202
Donkey anti-Mouse IgG (H+L) Highly Cross-Adsorbed Secondary Antibody, Alexa Fluor™ 555, 1:1000 IF, Invitrogen, # A-31570
Donkey anti-Rabbit IgG (H+L) Highly Cross-Adsorbed Secondary Antibody, Alexa Fluor™ 488, 1:1000 IF, Invitrogen, # A-21206
Donkey anti-Rabbit IgG (H+L) Highly Cross-Adsorbed Secondary Antibody, Alexa Fluor™ Plus 555, 1:1000 IF, Invitrogen, # A32794
Cy™3 AffiniPure Donkey Anti-Rabbit IgG (H+L), 1:1000 IF, Jackson ImmunoResearch Europe Ltd, #711-165-152
Cy™5 AffiniPure Donkey Anti-Rabbit IgG (H+L), 1:1000 IF, Jackson ImmunoResearch Europe Ltd, #711-175-152

Goat Anti-Mouse IgG (H + L)-HRP (horseradish peroxidase) Conjugate, Bio-Rad, 1:10 000 WB, #1706516
Goat Anti-Rabbit IgG (H + L)-HRP (horseradish peroxidase) Conjugate, Bio-Rad, 1:10 000 WB, #1706515

Validation

All antibodies used in this study were validated by the manufacturers or in previously published work from the lab (by ourselves using genetic knockout models when available or by using only secondary without primary antibodies).

Eukaryotic cell lines

Policy information about cell lines and Sex and Gender in Research

Cell line source(s)

C2C12 cell line. ATCC CRL-1772 lot: 70004012

Authentication

Commercial C2C12 cell line was authenticated by STR Profiling Analysis | ATCC

Mycoplasma contamination	Tested for being Mycoplasma free
Commonly misidentified lines (See ICLAC register)	No commonly misidentified cell line were used in this study.

Animals and other research organisms

Policy information about studies involving animals; ARRIVE guidelines recommended for reporting animal research, and Sex and Gender in Research

Laboratory animals	For in vivo experiments, both control C57 black 10 (C57BL10, The Jackson laboratory Bar Harbor, USA) and C57BL/10ScSn-Dmdmdx/J mdx were used (The Jackson laboratory Bar Harbor, USA) between 7 and 11 weeks. All animals were maintained under specific pathogen-free conditions. These mice were housed in a 12-h-light/12-h-dark cycle at a temperature controlled (23°C +/- 0.9°C) with 50%+/- 4% humidity, facility with free access to food and water.
Wild animals	This study did not involve wild animals.
Reporting on sex	We used only male mdx mice. Because, in humans, Duchenne muscular dystrophy is an X-linked neuromuscular recessive disorder affecting approximately 1 in 3,500 newborn males worldwide.
Field-collected samples	This study did not involve field-collected samples.
Ethics oversight	All procedures using animals were approved by the Institutional ethics committee and followed the guidelines of the National Research Council Guide for the care and use of laboratory animals and by the University of Ottawa Animal Care Committee. All procedures were in accordance with the Canadian Council of Animal Care Guidelines.

Note that full information on the approval of the study protocol must also be provided in the manuscript.

March 2021
